# Severe Heat Stroke Resuscitation Using a Body Bag in a Community Emergency Department

**DOI:** 10.7759/cureus.44045

**Published:** 2023-08-24

**Authors:** Karthik Pittala, Tyler F Willing, Charles C Worrilow, Matthew M Palilonis

**Affiliations:** 1 Department of Emergency and Hospital Medicine, Lehigh Valley Health Network/University of South Florida Morsani College of Medicine, Allentown, USA

**Keywords:** emergency medicine, hyperthermia, body bag, resuscitation, heat stroke

## Abstract

Heat stroke can lead to severe complications such as end-organ damage and death. The primary treatment modality for heat stroke is rapid cold-water immersion to lower the patient’s body temperature. This typically requires a large bath to place the patient in, which may not be available in small or community emergency departments. Although rarely present in the literature, a body bag for cold-water immersion can be used if a bath is not available. Here, we present a case of a 63-year-old male who presented to the emergency department unresponsive with hyperthermia after a heat wave warning was issued. After a thorough workup and imaging, the patient was given IV saline and naloxone, which did not improve his condition. Therefore, the patient was placed in a body bag filled with cold water and ice until his body temperature reduced to 100°F, after which he was removed and closely monitored. The patient was safely discharged and only required repeat lab work three days after discharge. This case highlights a unique technique that emergency physicians can utilize in scenarios where a typical cold-water immersion setup and execution are not possible.

## Introduction

Heat stroke is a life-threatening condition that is defined as an elevation in body temperature that is associated with central nervous system dysfunction [1]. There are two types of heat stroke: Classic heat stroke occurs in individuals during heat waves, while exertional heat stroke primarily occurs in athletes undergoing physical activity in hot environments [1]. Rapid cooling is a cornerstone of management, and cold-water immersion is one popular method (in addition to evaporative and convective cooling techniques, which are associated with lower mortality in cases of elderly non-exertional heat stroke) [2].

Cold-water immersion, however, can be difficult to implement in an emergency department (ED) due to acuity and lack of resources. Patient outcomes are shown to be directly related to the intensity and duration of hyperthermia in the literature, so rapid cooling is a necessity [2-4]. Without it, multiorgan system failure, disseminated intravascular coagulation (DIC), and death are examples of potentially severe complications of heat stroke that can arise [5,6]. This case details a patient who presented to the ED unresponsive and with a body temperature >104°F, thus meeting the heat stroke diagnostic criteria, who was cooled using cold-water immersion in a body bag with positive outcomes and eventual discharge a few days later.

## Case presentation

The patient is a 63-year-old male patient with a past medical history of depression, bipolar disorder, anxiety, gastroesophageal reflux disease (GERD), hypothyroidism, tobacco use disorder, alcohol use disorder, and recent chronic diarrhea who presented to the ED unresponsive and tachycardic after being dropped off by a work colleague. An initial temperature of 105.4°F temporal and 107.5°F rectal was measured, and blood pressure was 83/55 on the left arm and 87/51 on the right arm. The patient was incontinent of stool, unresponsive to pain, and unable to follow commands. The only pertinent history received from the work colleague was that he was working outside on a very hot day, and a heat wave warning was issued that day. The work colleague was unable to provide a history as he left the hospital immediately after dropping off the patient.

**Figure 1:**
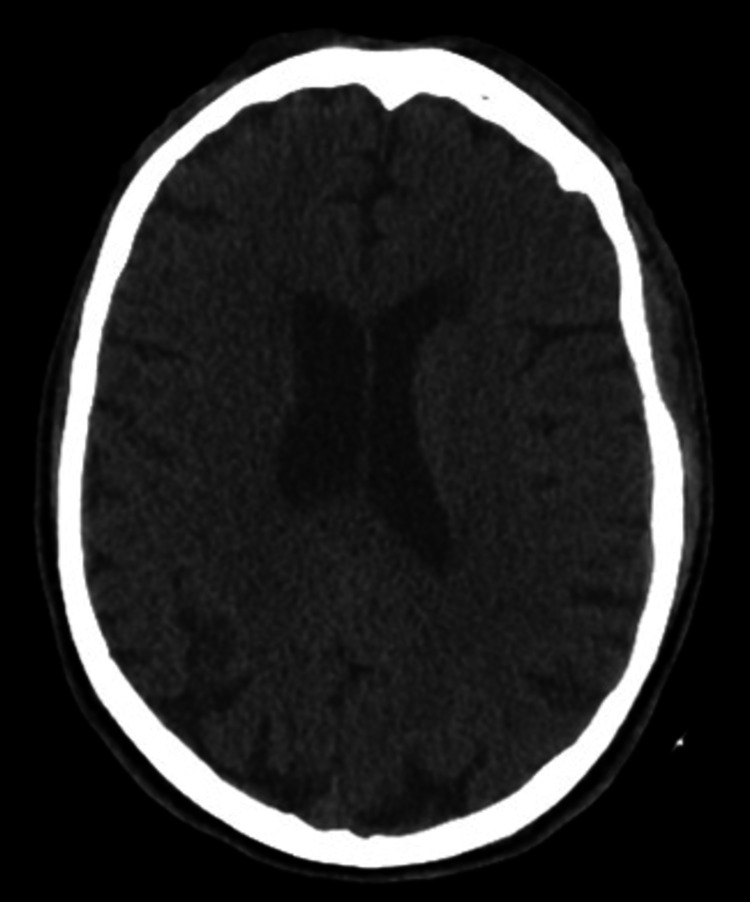
Normal head computerized tomography (CT) scan

**Figure 2:**
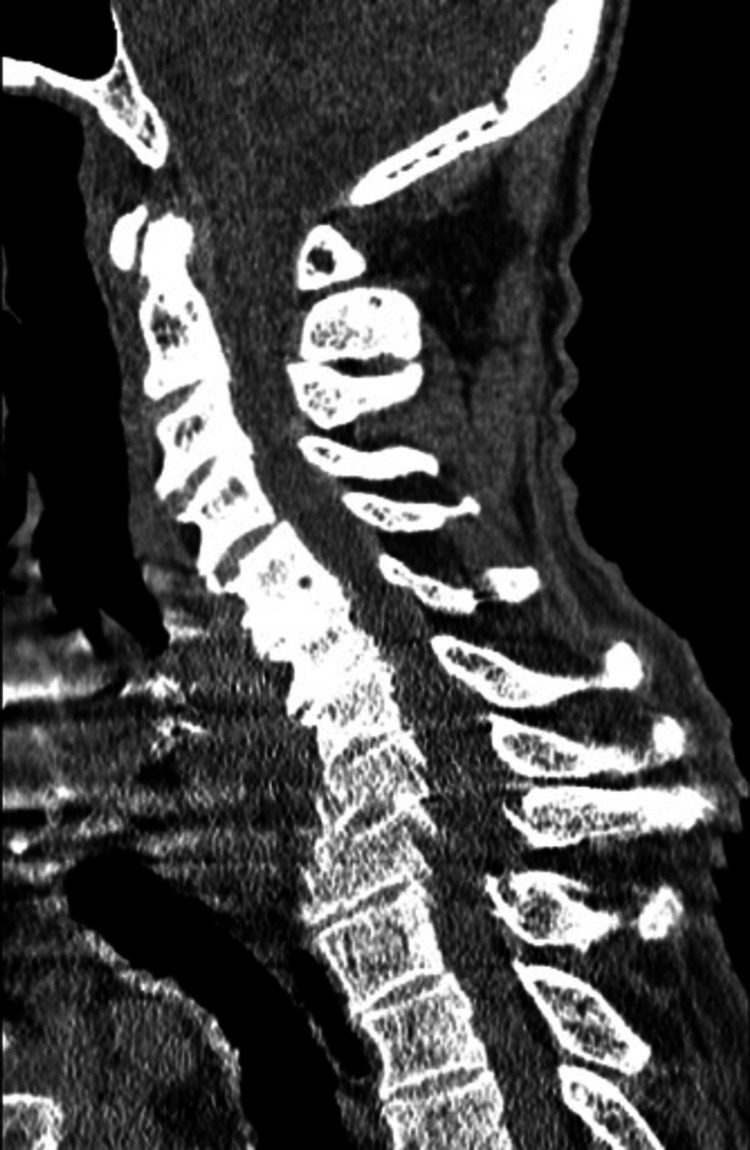
Normal cervical spine computerized tomography (CT) scan

**Figure 3:**
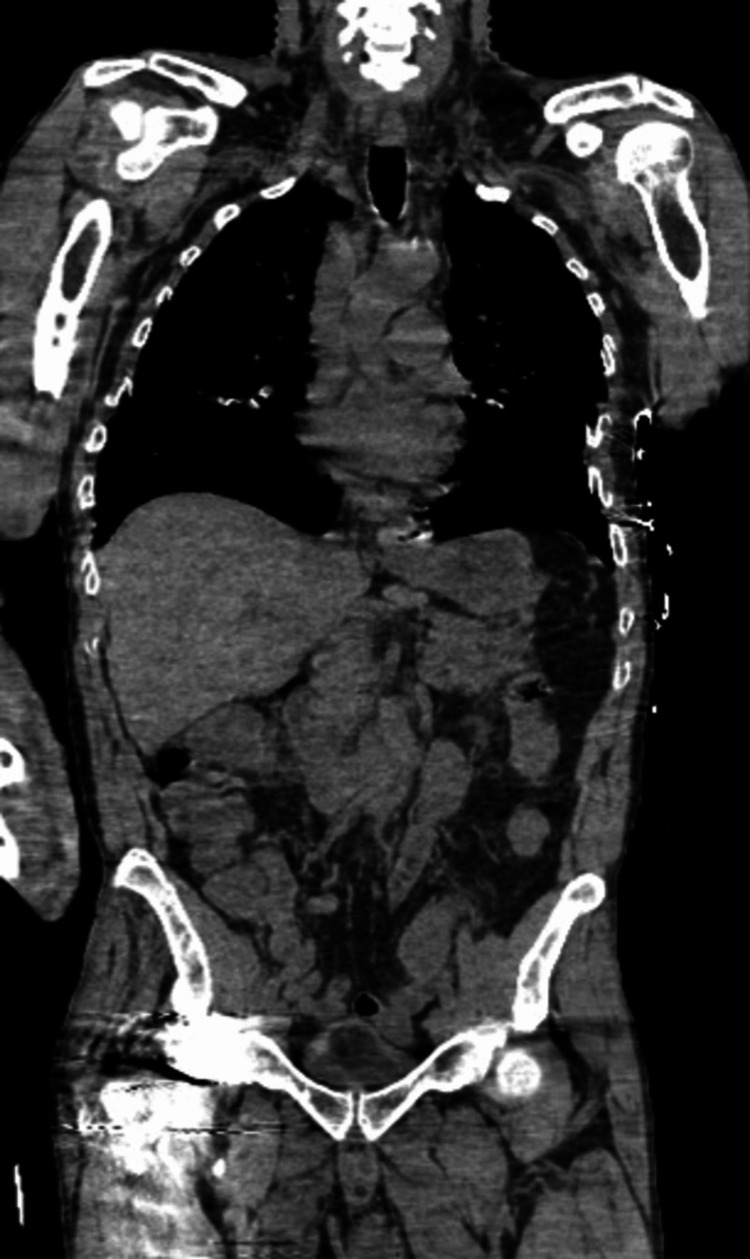
Normal chest/abdomen/pelvis computerized tomography (CT) scan

The patient was ill-appearing, unresponsive, and incontinent of feces on arrival. A cardiovascular examination showed sinus tachycardia. Pulses were 2+ and equal bilaterally for both upper and lower extremities. Glasgow coma scale (GCS) eye subscore was 1, verbal subscore was 1, and motor subscore was 4 for a total GCS score of 6. Though his initial GCS was 6, he quite quickly began to improve. The patient was protecting his airway upon arrival and had an end-tidal carbon dioxide monitoring in place measuring 36-38 mmHg. Therefore, an approach of cautious observation was used. Skin examination showed that the skin was very warm to touch without diaphoresis. A bruise was noted on the upper chest and an abrasion on the posterior left shoulder. On initial ED workup, pertinent abnormal labs included blood urine nitrogen (BUN) of 2.4 (7-28 mg/dL), creatinine of 2.64 (0.53-1.30 mg/dL), sodium of 133 (135-145 mmol/L), potassium of 2.4 (3.5-5.2 mmol/L), anion gap of 16 (3-11), eGFRcr of 26 (>59), white blood cell count (WBC) of 12.3 (4.0-10.5 thou/cmm), and urine analysis (UA) showing urine protein of 100 (negative mg/dL) and trace ketones. His CK and his blood sugar were normal. Computerized tomography (CT) scan head w/o contrast, CT cervical spine w/o contrast, and CT chest/abdomen/pelvis were all unremarkable as related to the patient’s current presentation (Figures 1-3).

The patient was given 4 L normal saline and 0.4 mg IV naloxone, which did not provide any relief or improvement in symptoms. The differential diagnosis included, among others, hypoglycemia, substance use hyperthyroidism, serotonin syndrome, and malignant neuroleptic syndrome. Some of these diagnoses either do not have testing or may have a diagnosis by tests that do not come back rapidly in the ED setting (for instance, thyroid tests). Given the climate, the signs, and symptoms that were most likely from severe heat stroke, the decision was made to rapidly cool the patient starting with ice packs placed on the patient's groin and axilla, and the setup for a cold-water immersion began. The patient was placed in a body bag on the stretcher laying on blankets to keep cold water around the patient, and then the body bag was filled with ice and water halfway. Staff kept telemetry cable and monitoring equipment dry on the front part of the patient’s torso to closely follow resuscitation efforts. His temperature was monitored with intermittent rectal temperatures. He continued to improve neurologically as his core temperature lowered, and he was able to sit up and state his name. Once the patient’s temperature had reduced to 100°F, the patient was removed from cold-water immersion. His mental status continued to slowly improve as his body temperature came down and GCS improved to 14 upon admission to the hospital. His drug screen and alcohol levels were negative. His creatinine improved to 2.07 mg/dL and potassium improved to 3.8 mmol/L. The patient’s status continued to improve outside of continuous low potassium, which required repletion prior to discharge. He was discharged safely and only required a repeat basic metabolic panel (BMP) in three days to follow potassium levels.

## Discussion

In this case, our differential diagnosis initially included, among others, hypoglycemia, substance use, trauma, or heat stroke. Because no trauma was reported (CT's negative), the extreme outside heat temperature along with the history of the patient working outside certainly swayed the team’s perception of the differential. Especially in the context of a normal drug screen, toxicology labs, ethanol, CK, and no improvement to naloxone, the treatment plan for heat stroke, were a natural evolution of limiting the differential. Heat stroke is a serious condition of thermoregulatory dysfunction that can result from either increased exposure to environmental heat or excessive physical exercise and is characterized by a core body temperature that rises above 104°F with altered mental status or central nervous system (CNS) dysfunction [6,7]. At such high temperatures, the body is gaining an excessive amount of heat that outpaces its ability to dissipate it [2]. Numerous methods of cooling are present in general clinical practice and in the literature that can be implemented in cases of heat stroke, but cold-water immersion (CWI) is consistently shown to be one of the most effective options [2,4,8]. Based on several meta-analyses and critical appraisals, CWI is the standard of care (when available) to perform on a hyperthermia patient, especially with neurological dysfunction [4,9-11]. The reason why CWI is so effective for rapid reduction in body temperature is because of the high conductance properties of water, which is 24-25 times greater than air [6,12].

In this case, a body bag was utilized to effectively reduce body temperature in a heat stroke patient in a setting where CWI in a bath would delay further care or not be possible due to a lack of resources. The use of a body bag ice bath is sparse in the literature, with only one case report noting that CWI in a body bag served to quickly cool the heat stroke patient and with complete resolution of her encephalopathy [12]. While this case report may not be generalizable, it serves as evidence that consideration for larger trials using this technology to provide proof of concept may be indicated.

## Conclusions

This case illustrates an important, real-life implementation of the body bag ice bath technique and proof of efficacy. This approach provides an additional tool for emergency medicine physicians to manage heat stroke patients. We aim for this case to further contribute to the treatment options for heat stroke and showcase a realistic option for community and rural EDs.
